# Hæmatocele, Perinterine

**Published:** 1874-05

**Authors:** T. G. Thomas


*THE MEDICAL AND SURGICAL REPORTER.—February* 7, 1874.

Clinic on Diseases of Women.—*By Prof. T. G. Thomas.—Hœmatocele.*—Last Christmas she went to church, and when there fainted, but rallied sufficiently to walk home. Since that time she has had severe pain in both of her sides, and a very severe pain in the back. At times she is feverish and has headache. These are all the symptoms the patient gives us, and the case is of interest from the little knowledge we obtain from that quarter.

*Vaginal Examination.*—When the finger is carried into the vagina we have a large mass pressing down posteriorly; the uterus itself is carried up anteriorly against the pubes. This mass blocks the vagina up. When it is pressed on we do not find it to be completely solid, which shows that in all probability it is not a fibroid tumor. There is a condition of affairs which it might be, though I think it is not, and that is an extra-uterine pregnancy. A tubal pregnacy might become dislocated and fall down into Douglass' cul-de-sac. If this were so it might be pushed up; this is firmly fixed.

Hæmatocele gives to the fingers the sensation of creaking when it is felt, as well as that of a sack full of fluid. The crucial test, which I do not propose to use here, because I feel so certain, is the hypodermic syringe. If it is a hæmatocele, a red fluid should be withdrawn, closely resembling cranberry jelly.

This disease is exceedingly likely to puzzle the practitioner. I know it has again and again puzzled me, and it cannot be tested with utter positiveness without having recourse to the crucial test I have mentioned.

When the blood effuses into the abdominal cavity and settles down into the cul-de-sac, a crust forms around it from coagulation of the fibrine on the periphery. Now it may occur, as indeed it has, that this may rupture and the blood again pass into the abdominal cavity, and cause peritonitis and death.

*Treatment.*—The treatment is exceedingly unsatisfactory.

You can, maybe, the more easily understand this when you consider the discussion which took place at the London Obstetrical Society, as to the merits of interference and non-interference. Dr. Barnes and Dr. Meadows were at issue on the subject. My own experience at present leads me to the view to let them alone. I did not always think so, as you will find from a case I will relate in which the aspirator was used. I was led to do this from another case which died from non-interference. I saw the patient and sent her to the Woman's Hospital. Not long after her admission, the House Surgeon sent me word that the patient was dying. At the postmortem it was found that the cause of the trouble was the rupture of the crust of the hæmatocele, allowing the escape of the contents into the cavity of the peritoneum. Not long after this I met the case I referred to first, and from the unfortunate issue in the case just mentioned, I inserted the aspirator needle and removed the contents of the hæmatocele. When the patient had been operated on, and the fluid removed, the friends were overflowing with gratitude, but in twenty-four hours the patient died in collapse. From the recital of both these cases you can understand the unfortunate difficulties in the way of treatment. I must say that this last case is the only one of the kind I ever had, and might be considered as not doing justice to the operation.

However, my own experience has decided me to let them alone, unless there is some urgent cause for the operation. In the case before us the chances are ninety out of a hundred that she will get well. While there is danger of eruption, there is greater danger, I think, in evacuating the contents. Should signs of septicæmia develop, the evacuation would then be decidedly indicated, and for this purpose the aspirator is undoubtedly the best.

